# Combining acquisition and image processing methods to improve evaluation of arial wall scar patterns after pulmonary vein isolation

**DOI:** 10.1186/1532-429X-18-S1-O20

**Published:** 2016-01-27

**Authors:** Adrian Lam, Erica Okene, Michael Lloyd, John Oshinski

**Affiliations:** 1 grid.213917.f0000000120974943Department of Biomedical Engineering, Georgia Institute of Technology, Atlanta, GA USA; 2 grid.189967.80000000419367398Division of Cardiology, Emory University, Atlanta, GA USA; 3 grid.189967.80000000419367398Department of Radiology, Emory University, Atlanta, GA USA

## Background

Pulmonary vein isolation (PVI) is a therapy to treat patients with atrial fibrillation (AF) but suffers a >30% recurrence rate. Studies have shown that gaps in the scar pattern encircling the pulmonary veins are linked to recurrence. Co-registering angiography images of the atrial blood pool to late gadolinium enhanced (LGE) images of the atrial wall to determine scar pattern suffer from differences in respiratory positions and resolution between the images. In addition, current image processing methods are cumbersome and require multiple viewing angles of the left atrial wall to assess the degree of encirclement. The goal of this study is to use a single imaging sequence twice: first to acquire angiographic images of the left atrium, followed by a second acquisition to acquire scar images of the atrial wall. We also present a novel visualization method to easily quantify the degree of scar encirclement.

## Methods

Seven patients who had undergone cryoballoon ablation 1-3 months prior received CMR at 3T (TimTrio, Siemens Healthcare, Erlangen, Germany). Contrast was injected at 0.3 mL/sec (0.2 mmol/kg) and a 3D navigator- and ECG-gated, IR-FLASH sequence was acquired 90 seconds after contrast injection for angiography images. Approximately 25 minutes after contrast injection, a Look-Locker sequence was acquired in the short-axis orientation to determine the optimal myocardial nulling point, and the same IR-FLASH sequence was run with a modified TI for LGE imaging.

**Figure 1:**
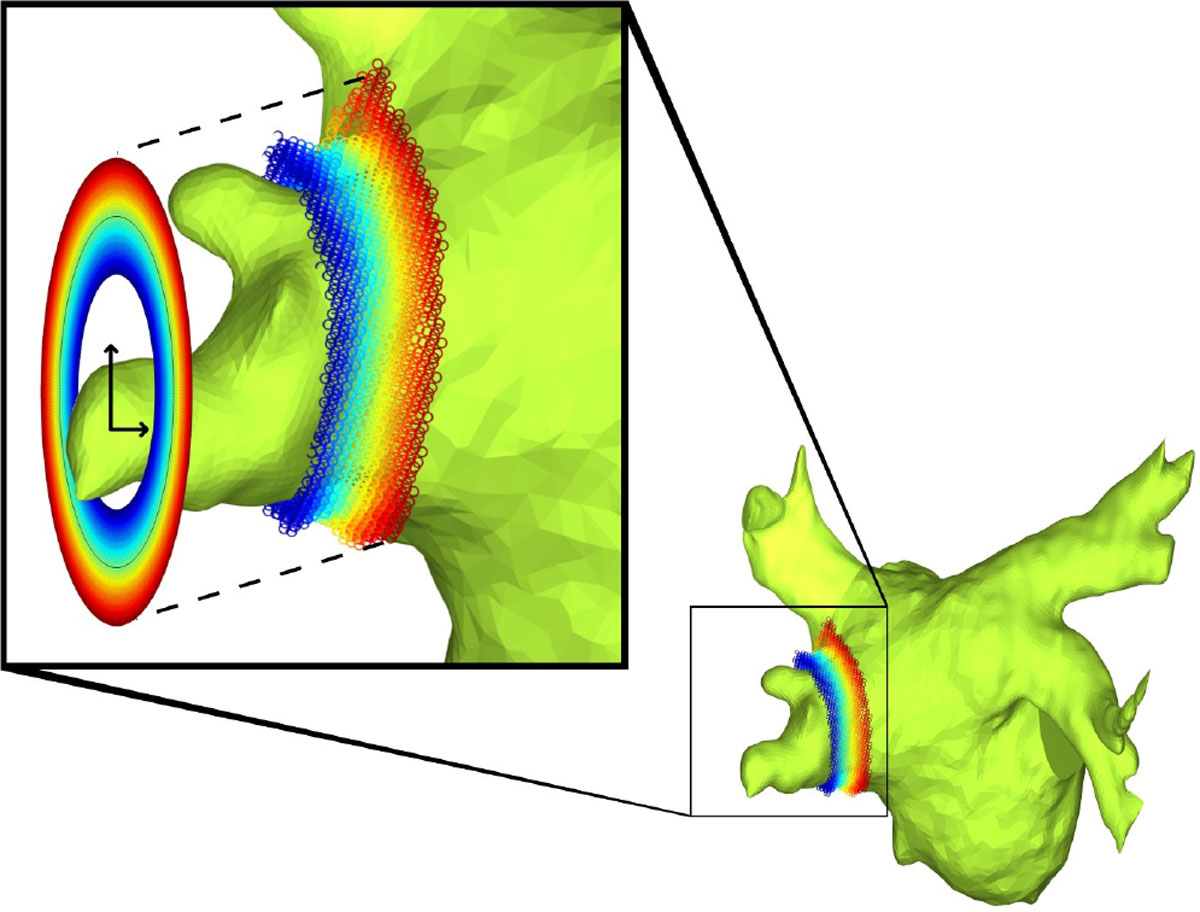
**Projection algorithm to convert atrial wall points to a pulmonary vein bullseye**. Points are projected outwards in the bullseye if closer to the atrial body and projected inwards if further along the pulmonary vein. The positive x-y axes on the bullseye represents the anatomical anterior-superior directions respectively.

Angiography and LGE images were co-registered using a gradient-ascent algorithm. The inner atrial wall was segmented on the angiography images, and borders were dilated 3 pixels to create the outer atrial wall. Borders were then transferred to LGE images to segment the atrial wall. A region of LV myocardium was sampled, and pixels with intensity > μ + 7σ were defined as *atrial wall scar*. Atrial wall points ± 5 mm above and below each pulmonary vein ostia were projected to a plane perpendicular to the pulmonary vein using a polar projection algorithm (Figure 1) to create four, 360° PV bullseyes for each patient. Scar encirclement was quantified by calculating the percent of scar about the bullseye.

## Results

**Figure 2:**
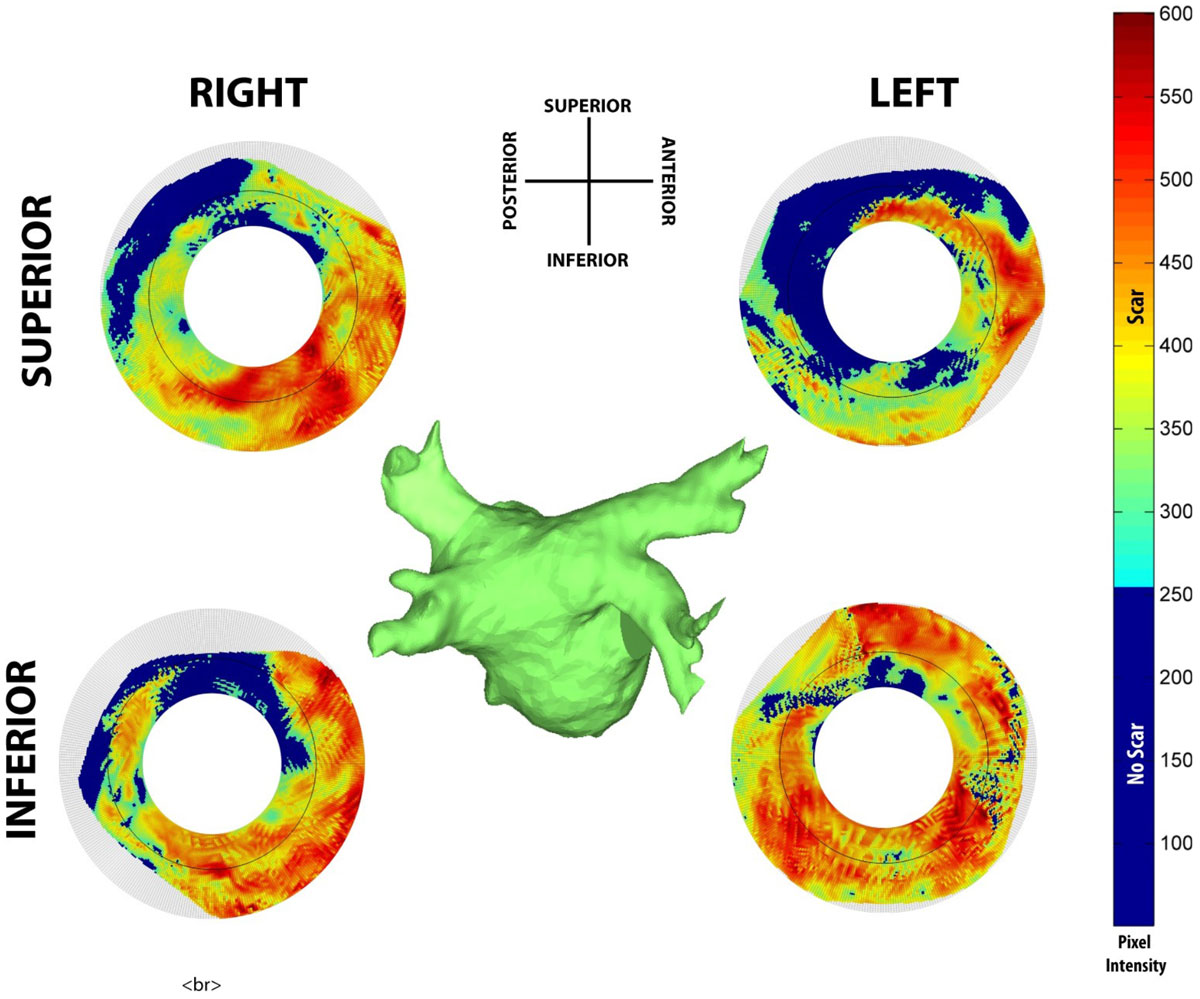
**Sample PV bullseyes are shown for the Left/Right (L/R) Superior/Inferior (S/I) veins**. Bullseye are thresholded for scar with blue representing non-scarred regions of atrial scar, while all other colors represent projected, interpolated atrial wall intensities. Incomplete encirclement can be seen for all veins except the RIPV.

Four pulmonary vein bullseyes were created for each patient (Figure 2). The average encirclement was 80 ± 12%, 88 ± 15%, 74 ± 17% and 67 ± 28% for the LSPV, LIPV, RSPV and RIPV respectively. A 120° segment of the anteroinferior portion of the LIPV of was found to be successfully ablated in all patients. In contrast, a 20° segment centered about the anteroinferior portion of the RIPV was found to be successfully ablated in only 2 patients.

## Conclusions

Acquisition of both angiographic and LGE images using the same sequences results in more simplified and accurate method for atrial wall segmentation. The use of PV bullseyes enhances visualization and enables simple quantification of pulmonary vein encirclement. The methodology can potentially be used to identify PV regions that are more difficult to isolate by cryoballoon ablation.

